# Cost-Effectiveness Analysis of Hepatic Arterial Infusion of FOLFOX Combined Sorafenib for Advanced Hepatocellular Carcinoma With Portal Vein Invasion

**DOI:** 10.3389/fonc.2021.562135

**Published:** 2021-03-09

**Authors:** Meiyue Li, Shen Lin, Leslie Wilson, Pinfang Huang, Hang Wang, Shubin Lai, Liangliang Dong, Xiongwei Xu, Xiuhua Weng

**Affiliations:** ^1^ Department of Pharmacy, First Affiliated Hospital of Fujian Medical University, Fuzhou, China; ^2^ Departments of Medicine and Pharmacy, University of California San Francisco, San Francisco, CA, United States; ^3^ Key Laboratory of Radiation Biology of Fujian Higher Education Institutions, First Affiliated Hospital of Fujian Medical University, Fuzhou, China

**Keywords:** cost-effectiveness analysis, hepatocellular carcinoma, combination therapy, sorafenib, hepatic arterial infusion chemotherapy, FOLFOX

## Abstract

**Background:**

Hepatic arterial infusion (HAI) of oxaliplatin, leucovorin, and fluorouracil (FOLFOX) plus sorafenib has a more desirable effect versus sorafenib for hepatocellular carcinoma (HCC) patients with portal vein invasion. However, considering the high cost of hepatic arterial infusion of chemotherapy (HAIC), this study evaluated the cost-effectiveness of HAIC plus sorafenib (SoraHAIC) versus standard care for HCC patients from the Chinese health system perspective.

**Methods:**

A Markov multi-state model was constructed to simulate the disease course and source consumption of SoraHAIC. Costs of primary therapeutic drugs were calculated based on the national bid price, and hepatic artery catheterization fee was collected from the Fujian Provincial Price Bureau. Clinical data, other costs, and utility values were extracted from references. Primary outcomes included life-years (LYs), quality-adjusted life years (QALYs) and incremental cost-effectiveness ratio (ICER). The robustness of model was verified by uncertainty sensitivity analyses.

**Results:**

SoraHAIC gained 1.18 QALYs (1.68 LYs) at a cost of $65,254, while the effectiveness and cost of sorafenib were 0.52 QALYs (0.79 LYs) and $14,280, respectively. The ICER of SoraHAIC vs sorafenib was $77,132/QALY ($57,153/LY). Parameter that most influenced the ICER was utility of PFS state. The probabilistic sensitivity analysis (PSA) showed that SoraHAIC was not cost-effective in the WTP threshold of 3*Gross Domestic Product (GDP) per capita of China ($30,492/QALY). But about 38.8% of the simulations were favorable to SoraHAIC at the WTP threshold of 3*GDP per capita of Beijing ($72,000/QALY). When 3*GDP per capita of Fujian ($47,285/QALY) and Gansu Province ($14,595/QALY) were used as WTP threshold, the acceptability of SoraHAIC was 0.3% and 0%, respectively.

**Conclusions:**

The study results indicated that SoraHAIC was not cost-effective in medium-, and low-income regions of China. In developed areas of China (Beijing), there was a 38.8% probability that the SoraHAIC regimen would be cost-effective.

## Introduction

Liver cancer, the sixth common human malignancies, ranks the fourth among all the cancer mortality in middle and high sociodemographic index (SDI) countries (1, 2). China accounts for more than half of the world’s confirmed cases of liver cancer (2, 3). It is reported that over 90% of cases are hepatocellular carcinoma (HCC), and approximately 12%–32% patients are diagnosed with portal vein invasion at the initial confirmed (4, 5). Currently, oral sorafenib is the first-line treatment for advanced HCC (6, 7). Nevertheless, the median progression-free survival (PFS) of patients with portal vein invasion regulated with sorafenib alone is only 2.6 months, and the effect prolonging the median overall survival (OS) is still weak (7, 8).

Repeating hepatic arterial infusions (HAI) with various chemotherapeutic regimens (such as cisplatin, oxaliplatin, 5-fluorouracil (5-FU), epirubicin, doxorubicin, and mitomycin-C) *via* an implantable port system has been reported as a valid therapeutic modality in treating unresectable HCC patients (9–13). Recently, a clinical trial showed that HAI of oxaliplatin, leucovorin, and fluorouracil (FOLFOX) combined with sorafenib in patients with advanced HCC have significantly prolonged OS than oral sorafenib (8). Based on this published positive clinical survival data, a phase-III trial performed in a Chinese setting, continued to assess the efficacy and safety among those receiving hepatic arterial infusion chemotherapy (HAIC) plus sorafenib (SoraHAIC) compared with those receiving sorafenib alone. The results showed combination therapy had a more favorable clinically significant outcome than sorafenib (OS:13.37 vs 7.13 months, *p* <.001; and PFS:7.03 vs 2.6 months, *p* <.001) (14).

At present, although SoraHAIC raises hope for patients extend survival, high prices may bring a heavy socioeconomic burden to patients and the healthcare system. In China, due to the enormous population of HCC patients, the limited medical resources and the unbalanced distribution of medical resources in different regions, pharmacoeconomic analysis of HCC treatment strategies are urgently needed to maximize the societal benefit. Until now, there has been no economic evaluation of HCC patients received SoraHAIC treatment. Our study was dedicated to compare the pharmacoeconomic of the two therapies from the perspective of the Chinese health system, using a Markov model and best available and transparent data.

## Methods

### Clinical Data

Medical information was derived from the NCT02774187 trial (14), which screened patients (meet the age of 18 years and above, histologically confirmed as HCC, etc.) and randomly assigned them to receive induction treatments sorafenib or SoraHAIC until disease progression (**Supplemental Figure 2**). The two groups of patients received 400 mg of sorafenib twice daily for 21 days in a cycle. After the HCC had progressed, some patients will cross over to receive oral sorafenib or HAIC, and the remaining patients will receive best supportive care (BSC) as second line therapy. Every patient received BSC after the second-line therapy failure. For the HAIC regimen, the patients underwent femoral artery puncture and tube placement at admission and the following FOLFOX regimen was administered *via* hepatic artery through a temporary port during a hospitalized period lasting 2 days:

- oxaliplatin, 85 mg/m^2^ of body surface area (BSA), day 1;
- leucovorin, 400 mg/m^2^ of BSA, day 1;
- fluorouracil, 400 mg/m^2^ of BSA, then 2,400 mg/m^2^ of BSA over 46 h on days 1 and 2^14^.

### Model Structure

A multi-state Markov model was constructed to simulate the costs and effectiveness of treatment of HCC with portal vein infusion plus standard treatment compared with standard treatment alone in China. TreeAge Pro 2017 (TreeAge Software, Williams-town, MA) was used to program the model and R software (version.3.6.1) to perform statistical analyses. The Kaplan-Meier survival curves were digitized for filtering the best fitting survival distribution. Ultimately, the Weibull survival distributions were used to generate the transition probabilities of the SoraHAIC and sorafenib strategies, respectively (**Table 1**).

**Table 1:** Relevant parameters of survival distribution.

Parameters	Value	Source
**Weibull survival model of PFS**		
SoraHAIC	Scale=0.0985, Shape=0.97, R^2^ = 0.9785399	(14)
Sorafenib	Scale=0.17, Shape=1.295, R^2^ = 0.9323149	(14)
**Weibull survival model of OS**		
SoraHAIC	Scale=0.02858, Shape=0.139235, R^2^ = 0.9814218	(14)
Sorafenib	Scale=0.06, Shape=1.48, R^2^ = 0.9722492	(14)

PFS, progression-free survival; OS, overall survival; SoraHAIC, sorafenib plus Hepatic Arterial Infusion of oxaliplatin, fluorouracil, and leucovorin.

The Markov model included four distinct and mutually exclusive heath states: PFS, recurrence-free survival (RFS), progressive disease (PD) and death (**Supplemental Figure 1**). In the model, cycle length was 21 days and time horizon (approximate 8 years) was determined by the expected time for 99% of the hypothetical patients modeled to die. During each cycle, the patients either remained in their assigned health state or progressed to a new health state, and were not allowed to return to previous health states. In the light of statistics from the National Bureau of Statistics of China, the background mortality rate was considered in the model (http://www.stats.gov.cn/tjsj/ndsj/2018/indexch.htm).

We made the following assumptions in the model: the PFS state was set as the initial health state of enrolled patients, either oral sorafenib alone or combination therapy was given to HCC patients, as specified in the clinical trial.

- After four cycles of induction therapies, 12.8% patients without progression in SoraHAIC group and 0.8% in sorafenib group underwent hepatectomy owing to down-staging. After surgery these patients entered the RFS health state period and were followed up regularly. According to the RECIST criteria in the NCT02774187 trial, when patients were identified with cancer recurrence after liver surgery, subsequent treatments (sorafenib alone, HAIC, and BSC) were used. Patients who did not receive surgical treatment continued receiving induction treatments until disease progression or until experiencing unacceptable toxic effects. As their disease progressed, subsequent treatments were the same as for the combined group. All patients continued to receive BSC after progression of second-line treatment, until death.
- Eventually, surgically treated patients also would be at risk of recurrence of HCC. The Milan criteria (MC) is the benchmark of recurrence risk for screening patients with liver cancer. who meet salvage treatment conditions for liver transplantation and resection. In general, most patients meet the MC standard for recurrence after hepatectomy. However, in the NCT02774187 trial, the HCC patients with portal vein invasion were beyond Milan standard. The clinical risk score (CRS) criteria have been proposed by researchers (15). CRS contains three risk factors including initial disease beyond MC, multinodular disease and presence of microvascular invasion, and each risk factor was assigned one point respectively. In the NCT02774187 trial, enrolled patients with HCC were accompanied by portal vein invasion, who at least had one point according CRS standard. Therefore, we used the CRS to estimate recurrence risk so as to predict cumulative recurrence incidences. Because patient recurrence risks after hepatectomy were not available, the CRS was assumed as 1 in our model. We used 5-year recurrence (33.5% probability of progression) estimates in our model (15–19), and we performed sensitivity analysis by using the recurrence incidences of 3 and 7-year (31.3% and 34.1%) as the upper and lower limits.

### Cost and Health Utility Estimates

Direct medical costs included the cost of the induction and subsequent treatments, examination (such as laboratory examination, computed tomography, and magnetic resonance imaging), hospitalization, hepatic artery catheterization, hepatectomy, treatment for grade 3–4 severe adverse events (SAEs) and BSC. The drug prices were derived from national bid price and hepatic artery catheterization fee was extracted from the Fujian Provincial Price Bureau. Other data were sourced from the published pharmacoeconomics literature (**Table 2**). Specifically, the costs of SAEs management were calculated only once in the first cycle, excepting the treatment of hand-foot syndrome (HFS), which throughout the entire life course of patients in our model. All costs were discounted by a 3% annual rate and converted to US dollars: $1 = RMB 6.77 in 2019.

**Table 2:** Base parameters input to model and ranges of sensitivity analysis.

Variable	Base Value	Range		Distribution	Source
		Min	Max		
**SoraHAIC: Incidence of AEs**					
Elevated ALT/AST	0.40	0.32	0.48	Beta	(14)
Neutropenia	0.097	0.077	0.12	Beta	(14)
HFS reaction	0.10	0.084	0.13	Beta	(14)
Diarrhea	0.089	0.071	0.11	Beta	(14)
Nausea/Vomiting	0.14	0.11	0.16	Beta	(14)
**Sorafenib: Incidence of AEs**					
Elevated ALT/AST	0.34	0.27	0.41	Beta	(14)
Neutropenia	0.025	0.02	0.03	Beta	(14)
HFS reaction	0.14	0.11	0.17	Beta	(14)
Diarrhea	0.12	0.099	0.15	Beta	(14)
Nausea/vomiting	0.025	0.02	0.03	Beta	(14)
**Cost per cycle, US $**					
Sorafenib	2308.32	1846.66	2769.98	Gamma	(20)
Oxaliplatin	273.31	218.65	327.97	Gamma	(20)
Fluorouracil	514.65	411.72	617.58	Gamma	(20)
Leucovorin	23.74	18.99	28.49	Gamma	(20)
HAIC	1817.03	1453.62	2180.44	Gamma	
Hepatectomy	8862.63	7090.10	10635.16	Gamma	(21)
Hospitalization	376.92	301.54	452.3	Gamma	(19)
Test	352.19	281.75	422.63	Gamma	(19)
BSC	357	167.64	847.84	Gamma	(22)
Elevated ALT/AST	42.54	33.04	49.56	Gamma	(2)
Neutropenia	82.39	65.91	98.87	Gamma	(23)
HFS reaction	11.54	9.23	11.54	Gamma	(24)
Diarrhea	5.66	4.53	6.79	Gamma	(25)
Nausea/Vomiting	48.35	38.68	58.02	Gamma	(23)
**Utility value**					
PFS	0.76	0.61	0.91	Beta	(2)
PD	0.68	0.54	0.82	Beta	(2)
**Disutilities**					
Elevated ALT/AST	0				(26)
Neutropenia	0.09	0.059	0.12	Beta	(27)
HFS reaction	0.016	0.013	0.019	Beta	(24)
Diarrhea	0.047	0.016	0.077	Beta	(28)
Nausea/Vomiting	0.048	0.038	0.058	Beta	(28)
**Proportion**					
Receiving hepatectomy after soraHAIC	0.128	0.10	0.15	Beta	(14)
Receiving hepatectomy after sorafenib	0.0082	0.0066	0.0098	Beta	(14)
Not receiving hepatectomy after soraHAIC	0.872	0.70	1.05	Beta	(14)
Not receiving hepatectomy after sorafenib	0.99	0.79	1.19	Beta	(14)
Receiving subsequent treatment on BSC after soraHAIC	0.41	0.33	0.49	Beta	(14)
Receiving subsequent treatment on BSC after sorafenib	0.33	0.26	0.39	Beta	(14)
Receiving subsequent treatment on HAIC after soraHAIC	0.29	0.23	0.35	Beta	(14)
Receiving subsequent treatment on HAIC after sorafenib	0.33	0.26	0.39	Beta	(14)
Receiving subsequent treatment on sorafenib after soraHAIC	0.3	0.24	0.36	Beta	(14)
Receiving subsequent treatment on sorafenib after sorafenib	0.35	0.28	0.42	Beta	(14)
BSA	1.72	1.38	2.06	Normal	(29)
Discount rate	3	0	5	Fixed	(30)
Recurrence rate	0.335	0.313	0.341	Beta	(15)

AEs, adverse events; ALT, alanine aminotransferase; AST, aspartate aminotransferase; HFS, hand-foot syndrome; BSC, best supportive care; PFS, progression-free survival; PD, progressive disease; BSA, body surface area; SoraHAIC, sorafenib plus Hepatic Arterial Infusion of oxaliplatin, fluorouracil, and leucovorin; HAIC, hepatic arterial infusion chemotherapy.

According to a published study in Chinese setting, we employed HCC utility values of 0.76 for all patients in the state of PFS and in RFS after surgery, and 0.68 for patients in the state of PD (2). Notably, the reduction of utility values and the occupation of other medical and health resources are closely related to SAEs (grade ≥3), five related SAEs (elevated ALT/AST, neutropenia, hand-foot skin reaction, diarrhea, nausea/vomiting) with higher incidence rates (≥5) were enrolled in the model. A sensitivity analysis was carried out on the uncertainty of the utility value.

### Main Outcomes

As shown in **Table 3**, quality-adjusted life-years (QALYs), life-years (LYs) and incremental cost-effectiveness ratio (ICER) were used to express the results. In the light of the World Health Organization (WHO) evaluation criteria, a particular regimen was deemed cost-effective when the ICER was below the WTP threshold, which was set as $30,492/QALY for China in 2019 (3*Gross Domestic Product (GDP) per capita) in this analysis.

**Table 3:** Summary of cost and effectiveness results in scenario.

Regimen	SoraHAIC	Sorafenib	Incremental
Overall cost ($)	65,254.07	14,280.30	50,973.77
Overall LYs	1.68	0.79	0.89
Total QALYs	1.18	0.52	0.66
ICER, ($)			
per LY			57,153.30
per QALY			77,132.51

LYs, life years; QALYs, quality-adjusted life years; ICER, incremental cost-effectiveness ratio; SoraHAIC, sorafenib plus Hepatic Arterial Infusion of oxaliplatin, fluorouracil, and leucovorin.

### Sensitivity Analyses

**Figure 1:**
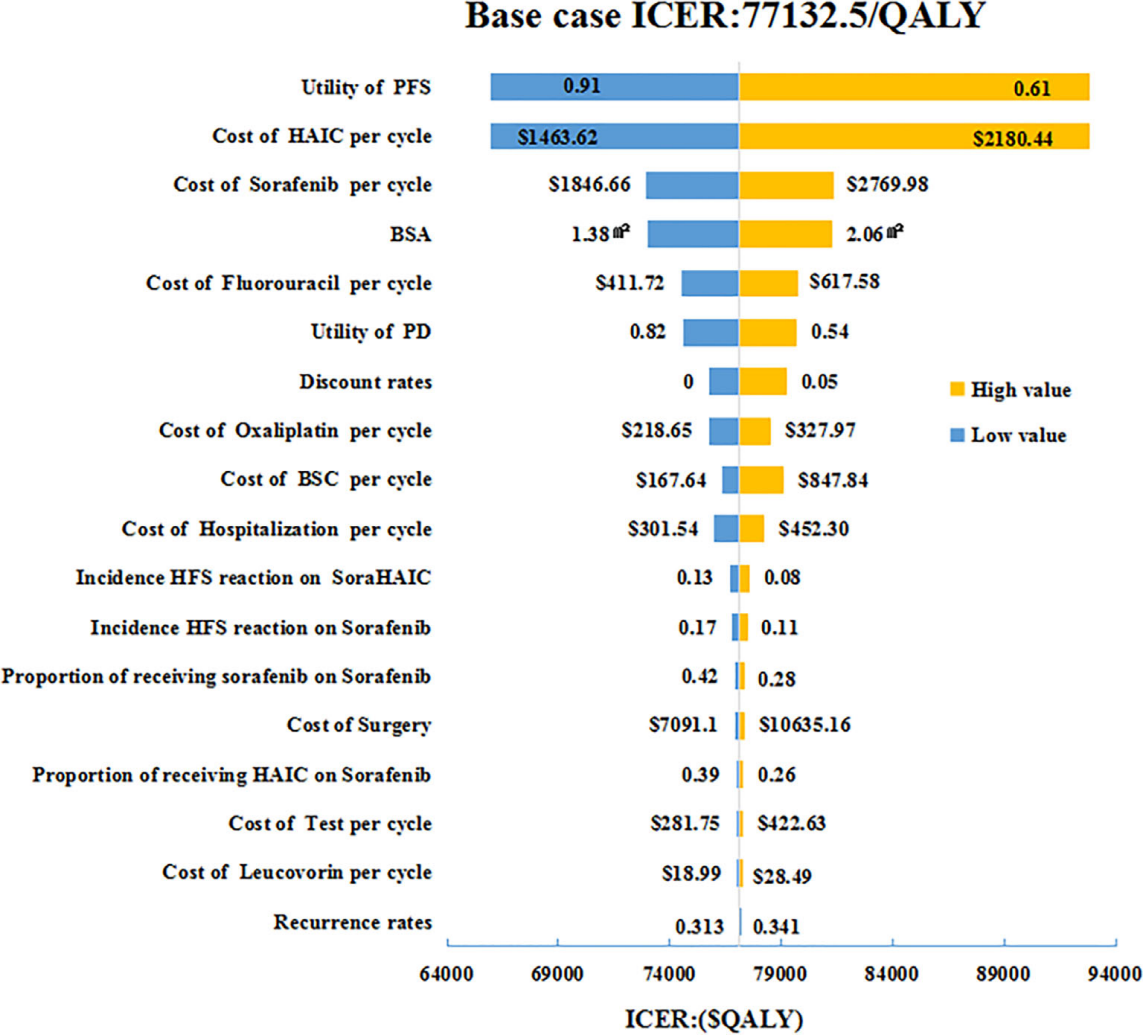
One-way sensitivity analyses of HAIC plus sorafenib in comparison with sorafenib in China. HAIC, hepatic arterial infusion chemotherapy; BSA, body surface area; BSC, best supportive care; HFS, hand foot syndrome; PFS, progression-free survival; PD, progressive disease; ICER, incremental cost-effectiveness ratio.

**Figure 2:**
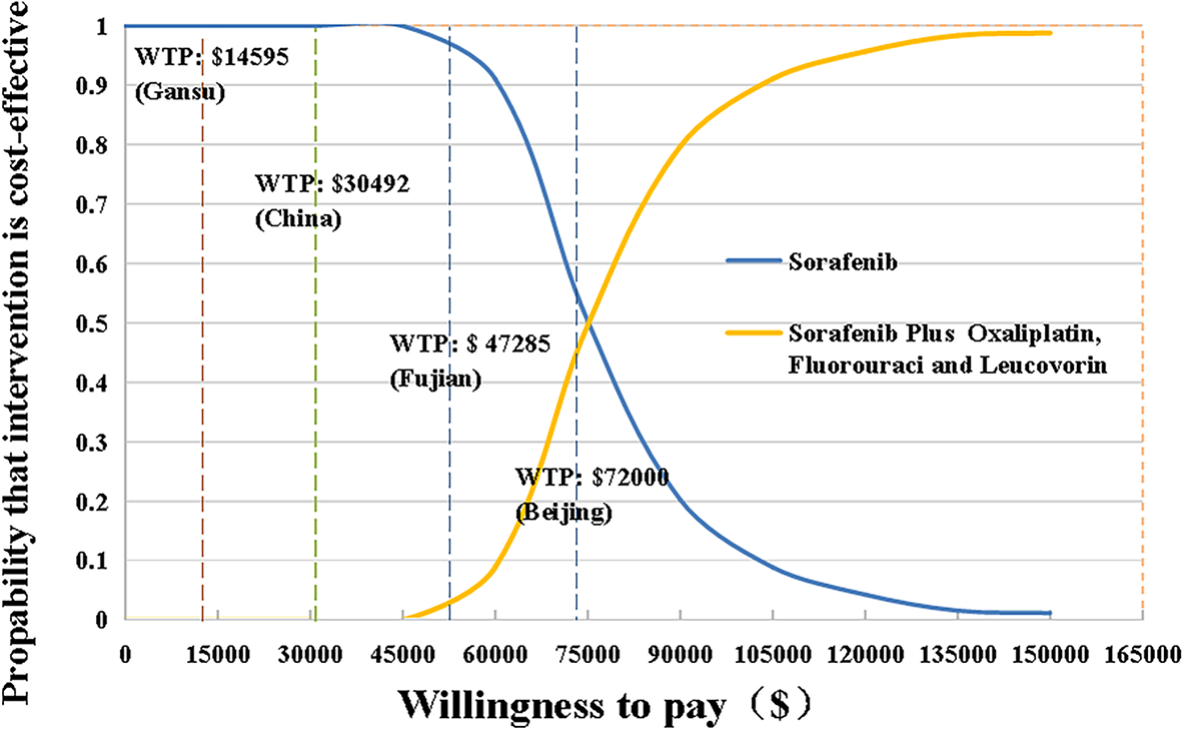
The cost-effectiveness acceptability curves for HAIC plus sorafenib strategy compared to the sorafenib strategy. HAIC, hepatic arterial infusion chemotherapy; WTP, willingness-to-pay.

**Figure 3:**
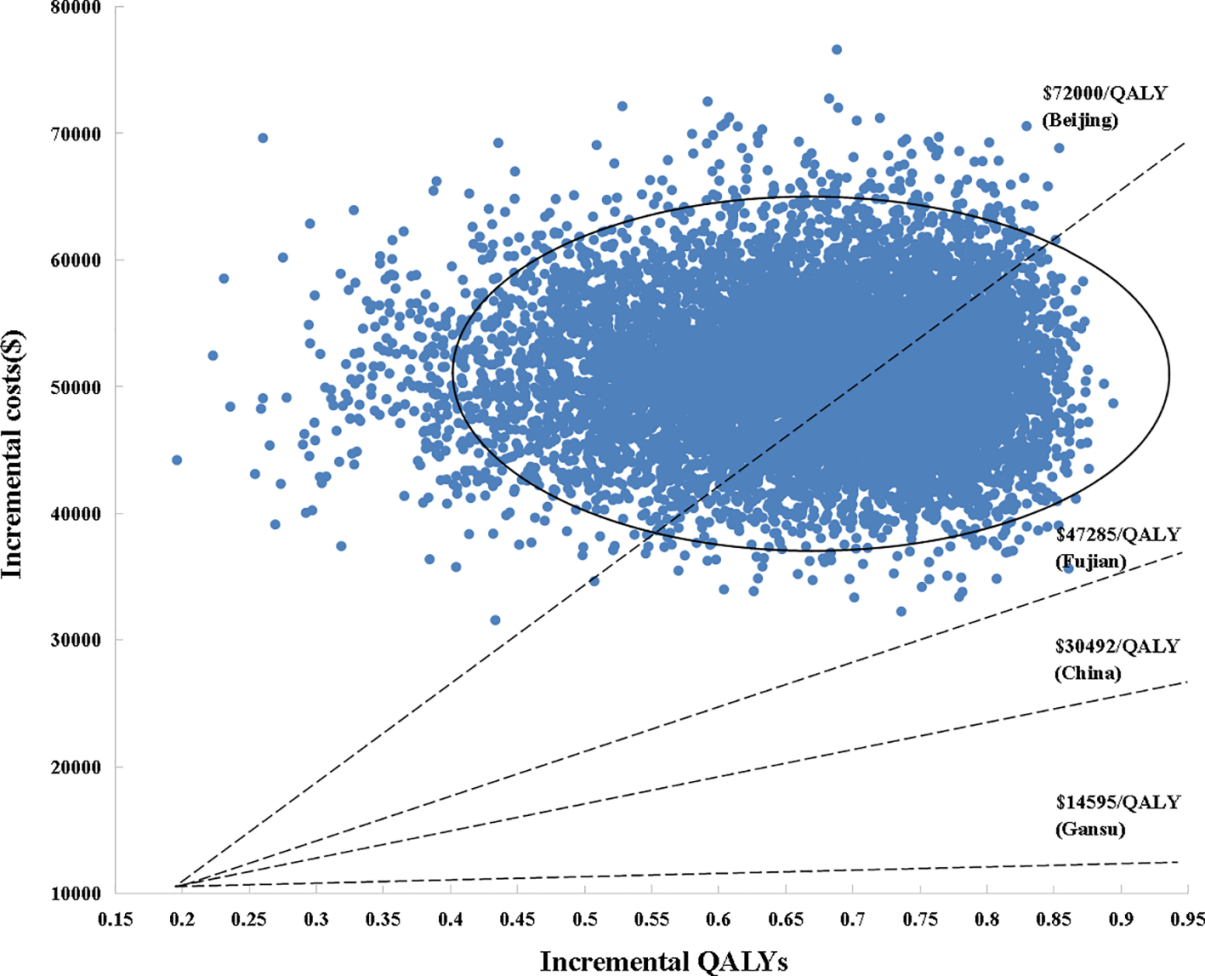
Scatter plot of probabilistic sensitivity analysis. Each point in the scatter plots corresponds to one sample of parameter values. QALYs, quality-adjusted life years; WTP, willingness-to-pay.

Sensitivity analyses were used to verify the robustness of model. One-way sensitivity analysis was presented in a tornado diagram and probabilistic sensitivity analysis (PSA) was presented in cost-effectiveness acceptability curves (CEACs) and a scatter plots, respectively (**Figures 1**–**3**). In one-way sensitivity analy sis, all the key variables were adjusted up and down within a reasonable range. The maximum and minimum values of these variables were obtained from the literature and a benchmark value ±20% was used in the case of lacking data. Particularly, the 3 and 7-year recurrence incidence of liver cancer after surgery were used to estimate upper and lower limits. The discount rate was varied from 0 to 5%. The PSA simulation involved Monte Carlo simulation of 10,000 repetitions, where each key parameter an appropriate distribution in the model was assigned. To be specific, utility values, probabilities or proportions were assigned beta distributions, BSA and costs were apportioned to normal distribution and gamma distributions, respectively.

## Results

### Base Case

For HCC patients, life expectancy was simulated over 8 years. SoraHAIC group achieved a 1.68 LYs, with 0.89 LYs more than standard treatment alone (**Table 3**). Accounting for QOL, SoraHAIC gained 1.18 QALYs with an additional 0.66 QALYs survival benefit for patients receiving sorafenib. Adding HAIC to sorafenib required extra expenditure about $50,974, which resulted in an ICER of $57,153/LY, and $77,132/QALY compared with sorafenib (**Table 3**). These ICER values were more than the WTP of 3*GDP per capita of China ($30,492/QALY), even beyond 3*GDP per capita of Beijing, which was the most developed region in China.

### Sensitivity Analyses

The tornado diagram (**Figure 1**) displayed the outcomes of one-way sensitivity analysis, which revealed that the model was more sensitive to utility value of PFS, and per cycle costs of HAIC and sorafenib. The ICER was always more than $30,492/QALY (3*GDP per capital) representing all of China, no matter how the key parameters alter within their specified range (**Figures 2**, **3**). Other variables, such as the other drugs costs, proportions receiving subsequent therapy, and recurrence incidence at 5-years had only minor effect on the ICERs (**Figure 1**).

The scatter plots of PSAs were shown in **Figure 3**. If we used the WTP threshold of $30,492/QALY in China, all the simulated points were above the set WTP threshold line in the Monte Carlo simulation of 10,000 repetitions. When the WTP threshold was established based on Beijing GDP per capita, SoraHAIC had a 38.8% probability to be cost-effective. On the contrary, when we set WTP based on Fujian and Gansu GDP per capita, all the simulated points were above the set WTP threshold line, which demonstrated that SoraHAIC was not cost-effective.

## Discussion

China is a major liver cancer country, accounting for 18% of the world’s population. Based on the latest and most complete data from the Chinese cancer registry, the incidence and mortality rates of liver cancer occupy 54.6% and 53.9% of the global total, respectively (3). The treatment of liver cancer accounts for an important share of cancer health expenditure. At present, the effectiveness of drug treatment of liver cancer is still unsatisfied. Researchers have been exploring new drugs or new ways of drug administration to improve the outcome (31). Preclinical studies suggested that SoraHAIC might have a synergistic effect (9, 10, 32). Furthermore, the rate of response to SoraHAIC (22%–48%) was significantly higher than that of systemic chemotherapy (8%–20.9%) or sorafenib alone (2%–3.3%) (4, 5, 14)

In the NCT02774187 trial, SoraHAIC demonstrated a relative increase in OS by 87.5% or 6.24 months compared with oral sorafenib among patients with HCC and portal vein invasion (14). Furthermore, SoraHAIC treatment shared an acceptable safety profile with sorafenib (14). Although potential use of HAIC raised hope for patients, its high price yielded a heavy financial burden on healthcare services and society. It was inevitable to explore the economic efficiency of HAIC given its considerable HCC attributable burden of disease in China. In addition, expenditure for public health in such a country like China which is both advanced and advancing must be apportioned for the best societal value. As far as we know, cost-effectiveness assessment on HAIC treatment regimens remained very few in literature, with only two pharmacoeconomic articles with contrasting conclusions on HAIC published from the U.S. perspective more than a decade ago (33, 34). Boris et al. suggested that systemic chemotherapy and HAIC were equally economic in terms of consuming health care resources to provide normal quality-adjusted survival times. Conversely, Romanus et al. proved that when the WTP threshold was $100,000/QALY, HAIC was not a cost-effective alternative compared to systemic chemotherapy. At present, there are no published economic studies evaluating the cost-effectiveness of SoraHAIC vs sorafenib in the treatment of HCC. Therefore, an economic evaluation carried out to determine the best choice for the HCC patient by considering both the effectiveness and the costs have great significance for health care decision making in middle-and low-income settings, especially for China which has a great number patients and a heavy medical burden.

In our study, compared with sorafenib alone, the ICER was much higher than the WTP threshold, suggesting that SoraHAIC treatment might not be a cost-effective option when viewed from the perspective of China as a whole. Another similar research study compared the economy of sorafenib in combination with transcatheter arterial chemoembolization (TACE) with sorafenib in China, which clearly pointed out that the ICER was over the WTP threshold and sorafenib plus TACE was also deemed uneconomical (31). The main possible reason was that these two treatments failed to reach cost-effectiveness threshold was likely due to the high costs to deliver treatments such as HAIC and TACE. Although the clinical OS effect had improved, it was still not economical. The utility of the PFS state was also an important factor in the model. In addition, there were additional surgical and drug treatment costs for those who do better across the two nations of groups; further adding to the costs of the more efficacious treatment. Importantly, there is a large gap in GDP per capita across China’s provinces/cities. In 2019, the data from National Bureau of Statistics showed that GDP per capita of Beijing, Fujian and Gansu are $24,000, $15,761, and $4,865, which represent the high- medium-, low-income regions respectively. Our results showed that SoraHAIC was not an economical regimen at the WTP of 3*GDP per capita of China. However, it could be concluded that the SoraHAIC group showed a 38.8% desire to be economical in the high-income region. In the medium-, low-income regions, the results were less economical (**Supplemental Table 1**). This led to the question that if there were different expectations of treatment accessibility across different regions of China, to match their differential GDP thresholds. Additionally, there was raised a question of fairness for treatment access across regions if these regional WTP thresholds were considered. There should be some method developed to rectify basing cost-effectiveness analysis (CEA) treatment thresholds on GDP when variation across regions were so great.

Published studies in U.S. indicated that cancer drugs were associated with more than 2-fold higher ICERs in comparison with non-cancer drugs. Both the majority of cancer and non-cancer WTP fell in the $100,000–150,000 or $50,000–100,000 ranges (35, 36). Most anticancer drugs focus on prolonging life, whose heightened value may support this higher WTP threshold per QALY and should be a topic for further research. We used the conservative three times of GDP per capita as the WTP threshold per QALY in conformity with the World Health Organization guidelines. If the WTP increased, our results would change and the SoraHAIC was more likely to be cost-effective. Under the trend of medical reform, China has invested a lot of vigor to address the outstanding problems in its medical system. The main problems of current medical and health care included the inequality of health care conditions across regions and the high financial burden faced by patients. Recognizing this, the “Healthy China 2020” initiative was proposed (37). Administration played a role of “strategic purchaser” of the medical insurance fund, and this could drastically lower the cost of medicines through various mechanisms such as multilateral negotiations, reductions in drug prices in exchange for volume, and others. On the other hand, the costs of other factors rather than just drug prices should be considered such as the special drugs delivery systems, like HAIC, TACE, which were studied here. These factors also added financial burden to patients along with their life saving value, and these are often ignored by the public and decision makers. So our work would evoke renewed attention to the importance of the economics of special drug administration routes, especially for HCC. Annually, it is estimated that 460,000 patients are diagnosed as HCC in China, and about 420,000 of them have died from HCC (19). If those approximately 12% patients in HCC with portal vein invasion can receive the benefit of HAIC treatment, and it will make a huge contribution to Chinese society cancer survival.

An important strength in our research was that we adopted the risk of HCC recurrence after resection to build a more accurate model. Based on the CRS created to predict cumulative incidences of recurrence beyond MC, patients with a score of 0, 1, 2 and 3 had recurrence rates of 18.7%, 33.5%, 48.5%, and 67.1% cumulatively across 5-year, respectively (30). Notably, in the light of the CRS criteria, combined with the characteristics of the enrolled patients in the NCT02774187 experiment, postoperative patients were regarded as having a CRS score of 1 and a 5-year recurrence rate of liver cancer of 33.5% in our model.

It should be noted that, our analysis had several limitations. First, the hepatic artery catheterization fee was acquired from the Fujian Provincial Price Bureau due to the lack of unified price across China. Although it might cause some bias, the sensitivity analysis demonstrated it did not result in a reversal result. Second, according to the comprehensive NCT02774187 trial, when patients developed disease progression or unacceptable toxicity after first-line treatment, more than 50% of patients received sorafenib and SoraHAIC as their second-line regimens. Regorafenib, cabozantinib, nivolumab, and Pembrolizumab have been approved by China Food and Drug Administration (CFDA) for second-line treatment of patients with advanced HCC in China. However, lacking of survival data for enrolled patients received the above-mentioned medications was a barrier for further analysis. In addition, in our model, the additional cost and utility values were sourced from the published literature, which didn’t provide more demographic information. If relevant data are available in the future, the analysis could be carried out correspondingly. Third, due to the lack of the utility values data of patients in SoraHAIC group in the PFS and PD stage, we used the same health utility value of HCC for two treatment strategies and adjusted the utility scores with SAEs≥3. Meanwhile, there was a brief decrease to the utility values after the first few days of catheterization. Actually, we excluded this transient change in the model due to the lack of an accurate disutility value and the disutility caused by catheterization was less than 2 days, thus might produce only subtle impact on our model. Furthermore, the underestimated disutility of HAIC would result in a better QALYs in our base case result than reality. In reality situation, the lower QALYs of SoraHAIC would rise the ICER and strengthen our uneconomic result instead of changing it. Fourth, modeling with the Weibull function to fit the long-term OS survival of HCC patients became an unavoidable limitation of this study. With more mature fitting methods available, the model could be validated against the actual long-term survival data in future. Finally, unlike western countries, hepatitis B virus is the main etiology of HCC than hepatitis C virus in China (14), so whether our finding was suitable for Western countries or not is still an open question. Nevertheless, our research still reflects the current treatment condition of HCC patients in China, which has brought an unbearable economic burden to the patients, families, and the medical system. Therefore, we considered that the results of this study could provide an effective reference for clinical and policy decision makers in China and added to the conversation of how regional differences in GDP based thresholds should be used in determinations of cost-effectiveness of different cancer treatments.

## Conclusions

From the perspective of Chinese health system, SoraHAIC was estimated not cost-effective vs sorafenib for the treatment of HCC patients with portal vein invasion at a WTP threshold of $30,492/QALY in China. When considered economic differences of regions, SoraHAIC was not cost-effective in medium-, low-income regions, but it showed litter favorable in developed areas of China (Beijing). Our findings suggested that clinicians and policy makers should take cautious to interpret our results with consideration of regional difference. Further discussion is warranted when selecting appropriate WTP thresholds for cost-effectiveness of cancer treatments.

## Data Availability Statement

The raw data supporting the conclusions of this article will be made available by the authors, without undue reservation.

## Author Contributions

All authors contributed to the study conception and design. Propose concepts, analyze statistics, and write the manuscript were performed by ML, SL, and XW. Study selection and data extraction were done by ML, SL, PH, HW, and SBL. LW, XX, and XW commented on previous versions of the manuscript. All authors contributed to the article and approved the submitted version.

## Funding

This work was supported by the National Natural Science Foundation of China (81973473), the Natural Science Foundation of Fujian Province (2019J01446), and the Startup Fund for Scientific Research, Fujian Medical University (2018QH1091).

## Conflict of Interest

The authors declare that the research was conducted in the absence of any commercial or financial relationships that could be construed as a potential conflict of interest.

## Supplementary Material

Supplementary Figure 1 Decision-analytic model considering various health state.

Supplementary Figure 2 Baseline characteristics of the intention-to-treat population. SD, standard deviation; IQR, interquartile range; ECOG PS, Eastern Cooperative Oncology Group Performance Status; HBV, hepatitis B virus; HCV, hepatitis C virus; AFP, alpha-fetoprotein; Alb, albumin; Tbil, total bilirubin; Vp4, main portal vein invasion; Vp3, first branch portal vein invasion; Vp2, second branch portal vein invasion; Vp1, third branch portal vein invasion. Supplement Figure S**(A), (B), (C), (D)** Simulated weibull survival curve and cohort study curve.

Supplementary Table 1 The cost-effective probability in Monte Carlo 10,000 simulations in different GDP per capita. GDP, Gross Domestic Product.

## Abbreviations

AEs, adverse events; ALT, alanine aminotransferase; AST, aspartate aminotransferase; BSA, body surface area; BSC, best supportive care; CEA, cost-effectiveness analysis; CEACs, cost-effectiveness acceptability curves; CRS, clinical risk score; FOLFOX, oxaliplatin, leucovorin and fluorouracil; GDP, Gross Domestic Product; HAI, hepatic arterial infusion; HAIC, hepatic arterial infusion of chemotherapy; HCC, hepatocellular carcinoma; HFS, hand-foot syndrome; ICER, incremental cost-effectiveness ratio; LYs, life-years; MC, Milan criteria; OS, overall survival; PD, progressive disease; PFS, progression-free survival; PSA, probabilistic sensitivity analysis; QALYs, quality-adjusted life years; RFS, recurrence-free survival; SAEs, severe adverse events; SDI, sociodemographic index; SoraHAIC, HAIC plus sorafenib; TACE, transcatheter arterial chemoembolization; WHO, World Health Organization; 5-FU, 5-fluorouracil.
