# Supplementary figures and images for: Cost-Effectiveness Analysis of Hepatic Arterial Infusion of FOLFOX Combined Sorafenib for Advanced Hepatocellular Carcinoma With Portal Vein Invasion
Source: Front Oncol. 2021 Mar 9;11:562135. doi: 10.3389/fonc.2021.562135 (PMC7985441; doi:10.3389/fonc.2021.562135)

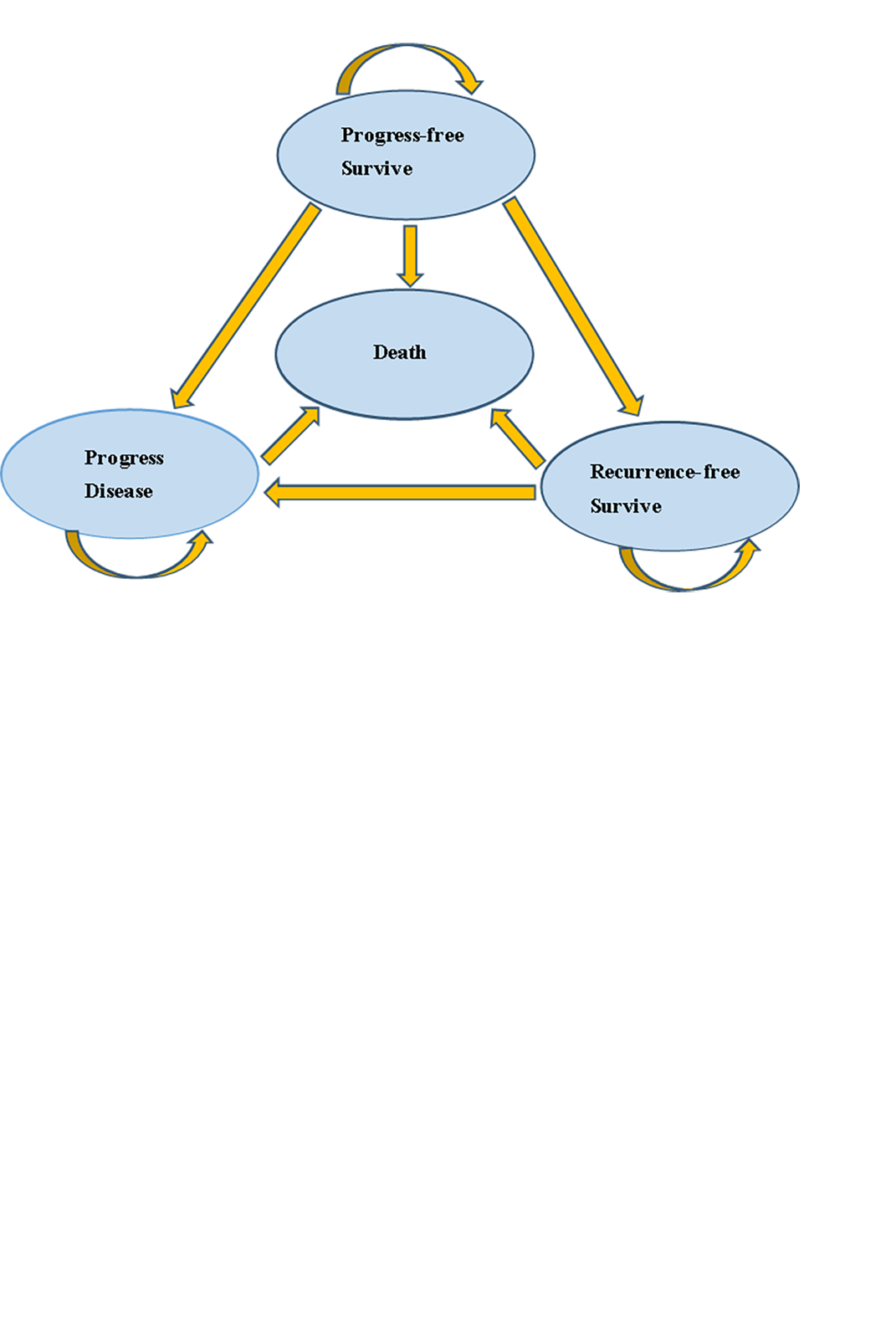

Supplement: Supplementary Figure 1 — Decision-analytic model considering various health state. [file Image_1.tif]

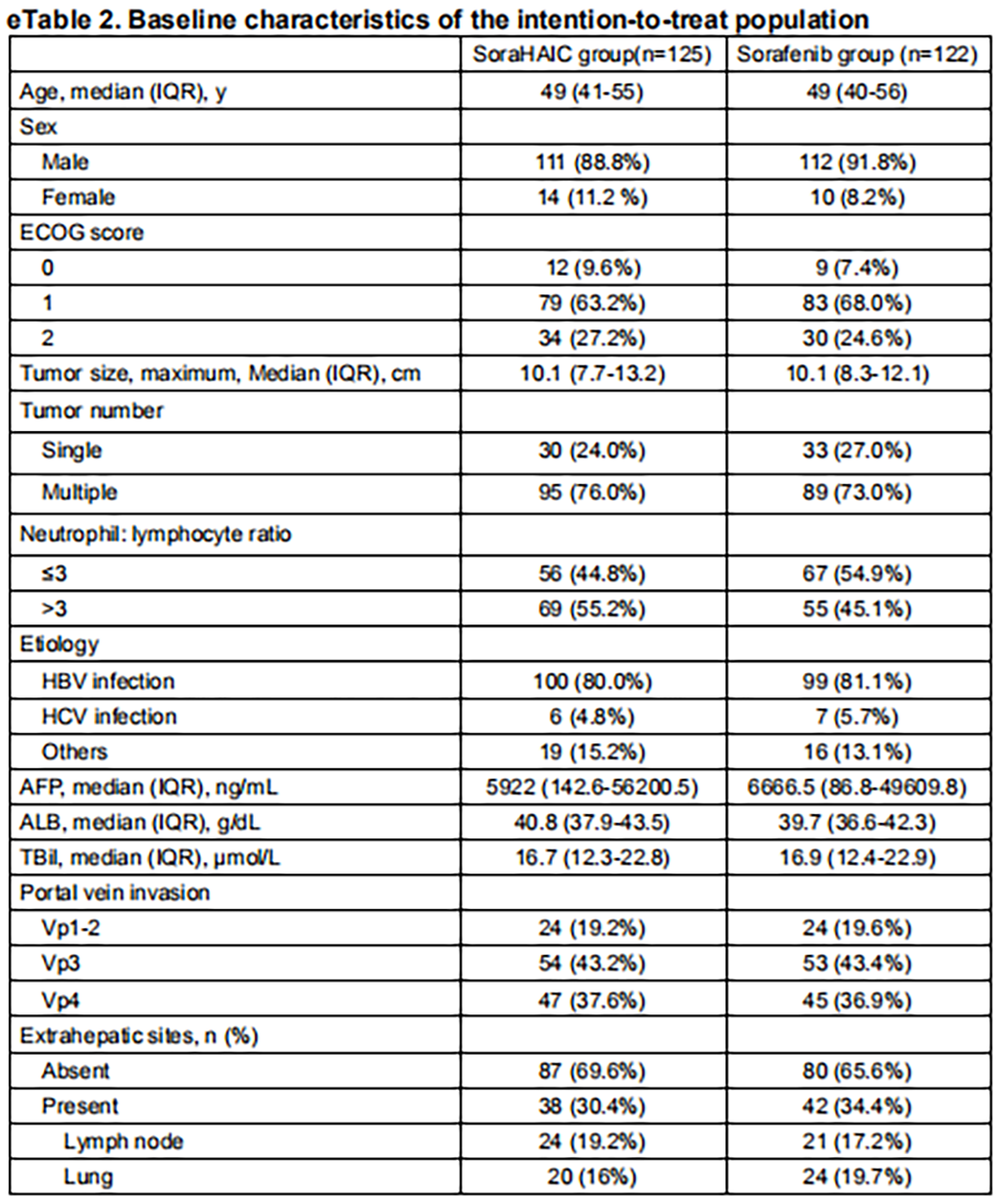

Supplement: Supplementary Figure 2 — Baseline characteristics of the intention-to-treat population. SD, standard deviation; IQR, interquartile range; ECOG PS, Eastern Cooperative Oncology Group Performance Status; HBV, hepatitis B virus; HCV, hepatitis C virus; AFP, alpha-fetoprotein; Alb, albumin; Tbil, total bilirubin; Vp4, main portal vein invasion; Vp3, first branch portal vein invasion; Vp2, second branch portal vein invasion; Vp1, third branch portal vein invasion. Supplement Figure S(A), (B), (C), (D) Simulated weibull survival curve and cohort study curve. [file Image_2.tif]

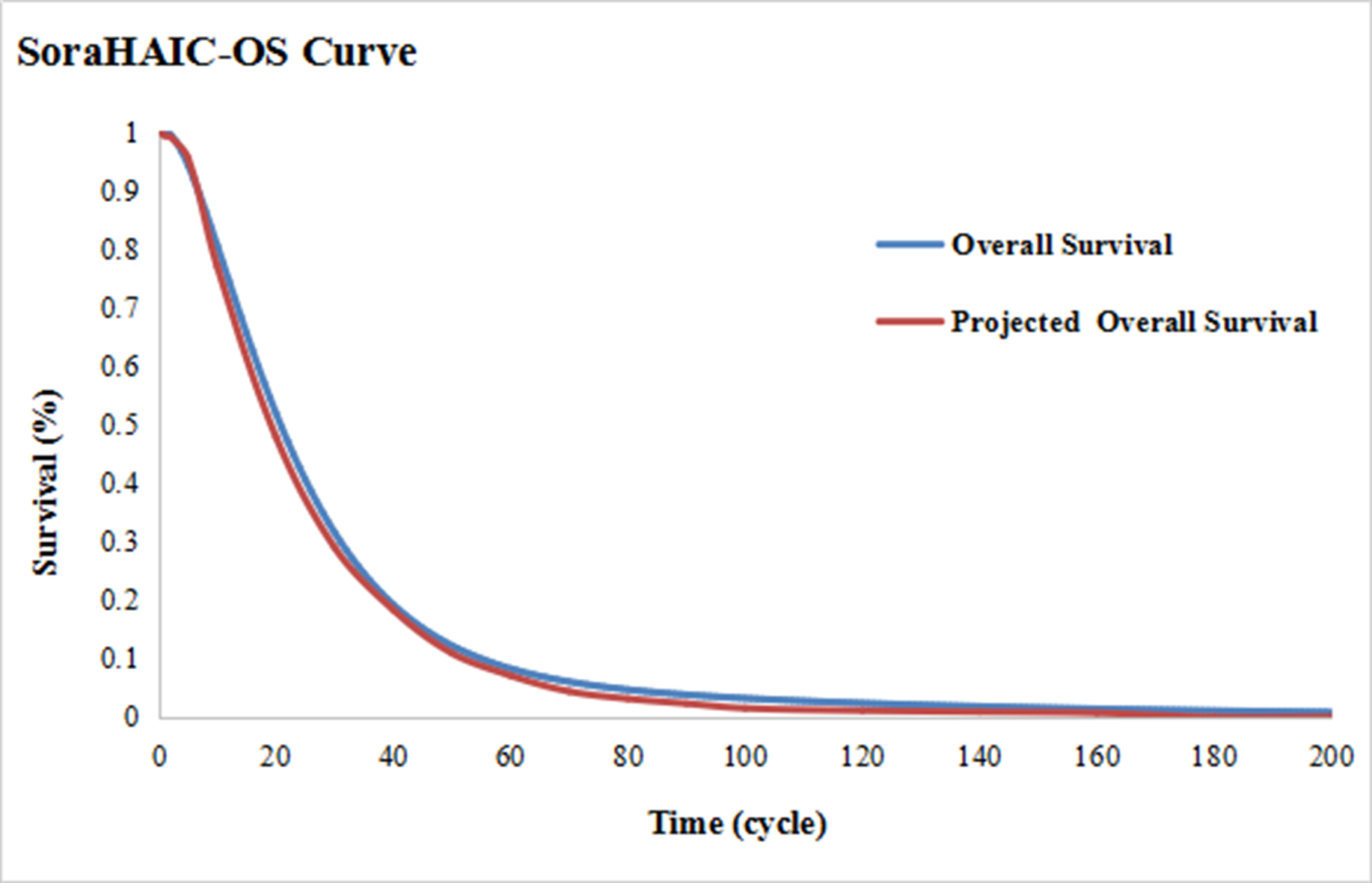

Supplement: Supplementary file 3 [file Image_3.tif]

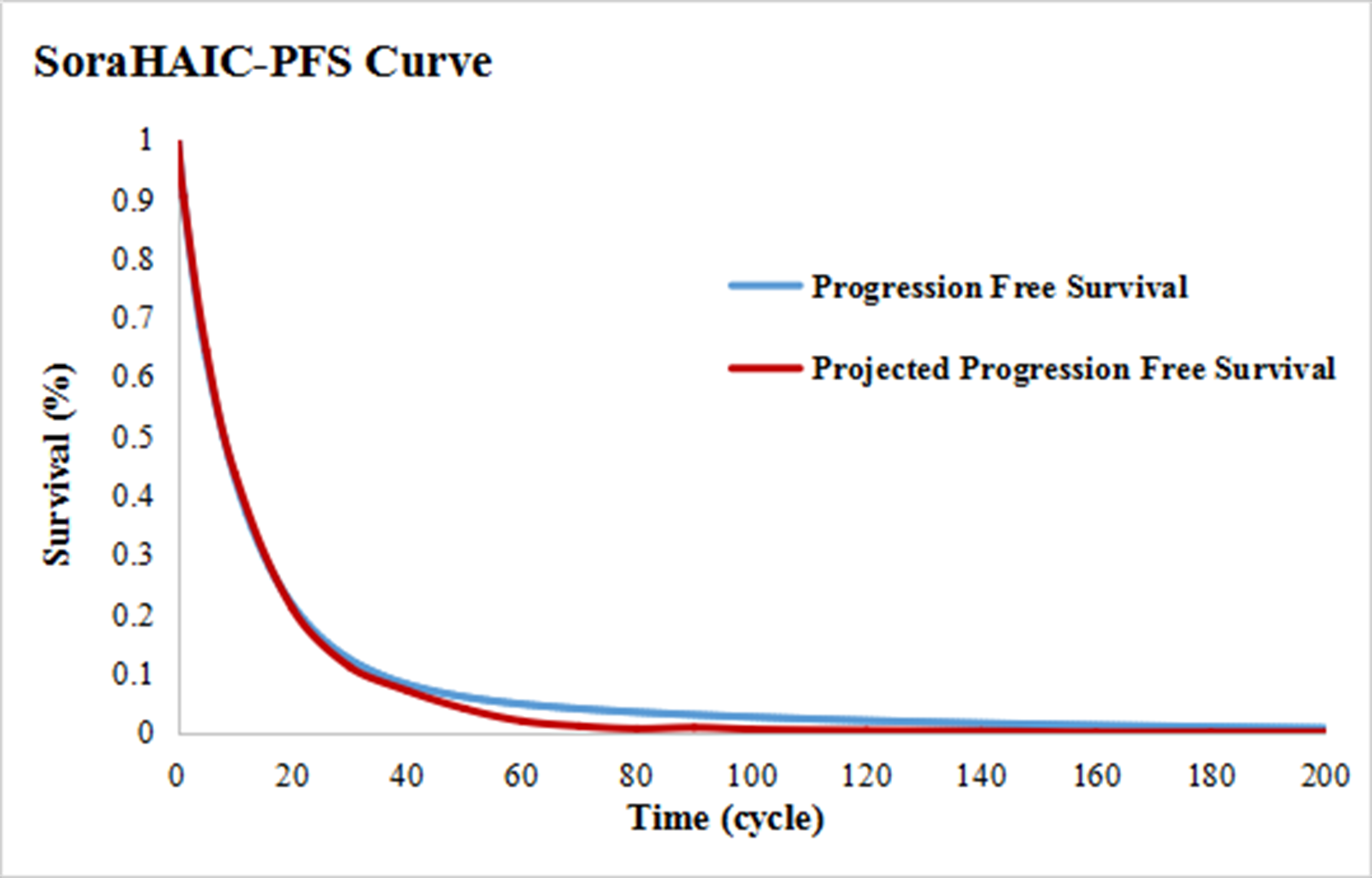

Supplement: Supplementary file 4 [file Image_4.tif]

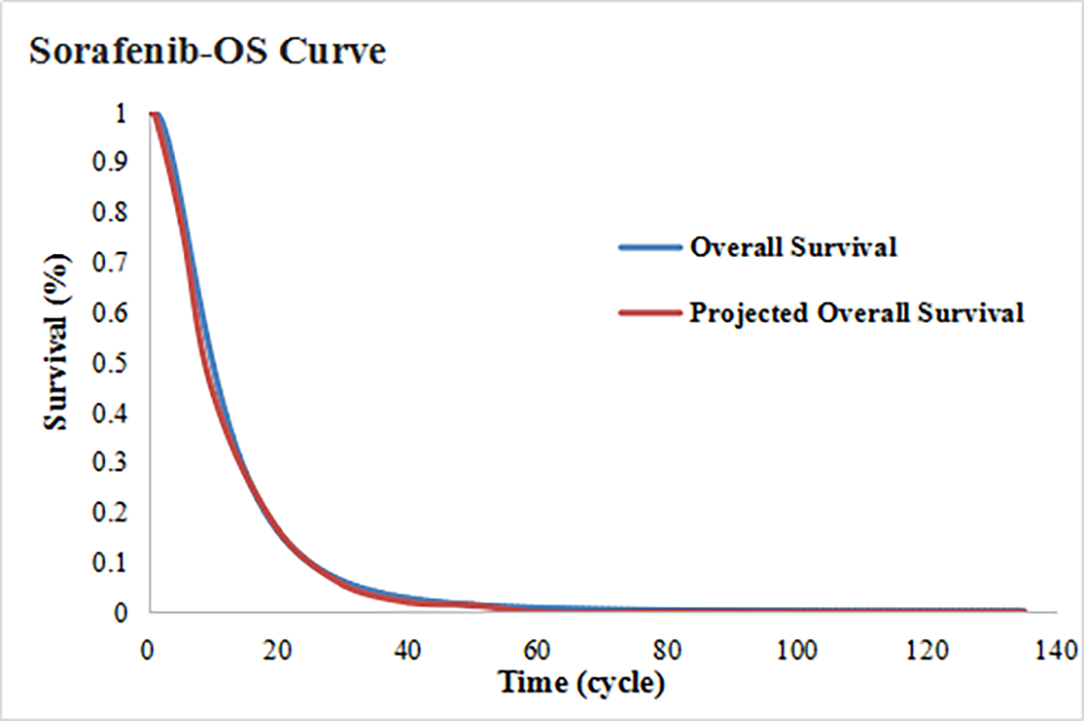

Supplement: Supplementary file 5 [file Image_5.tif]

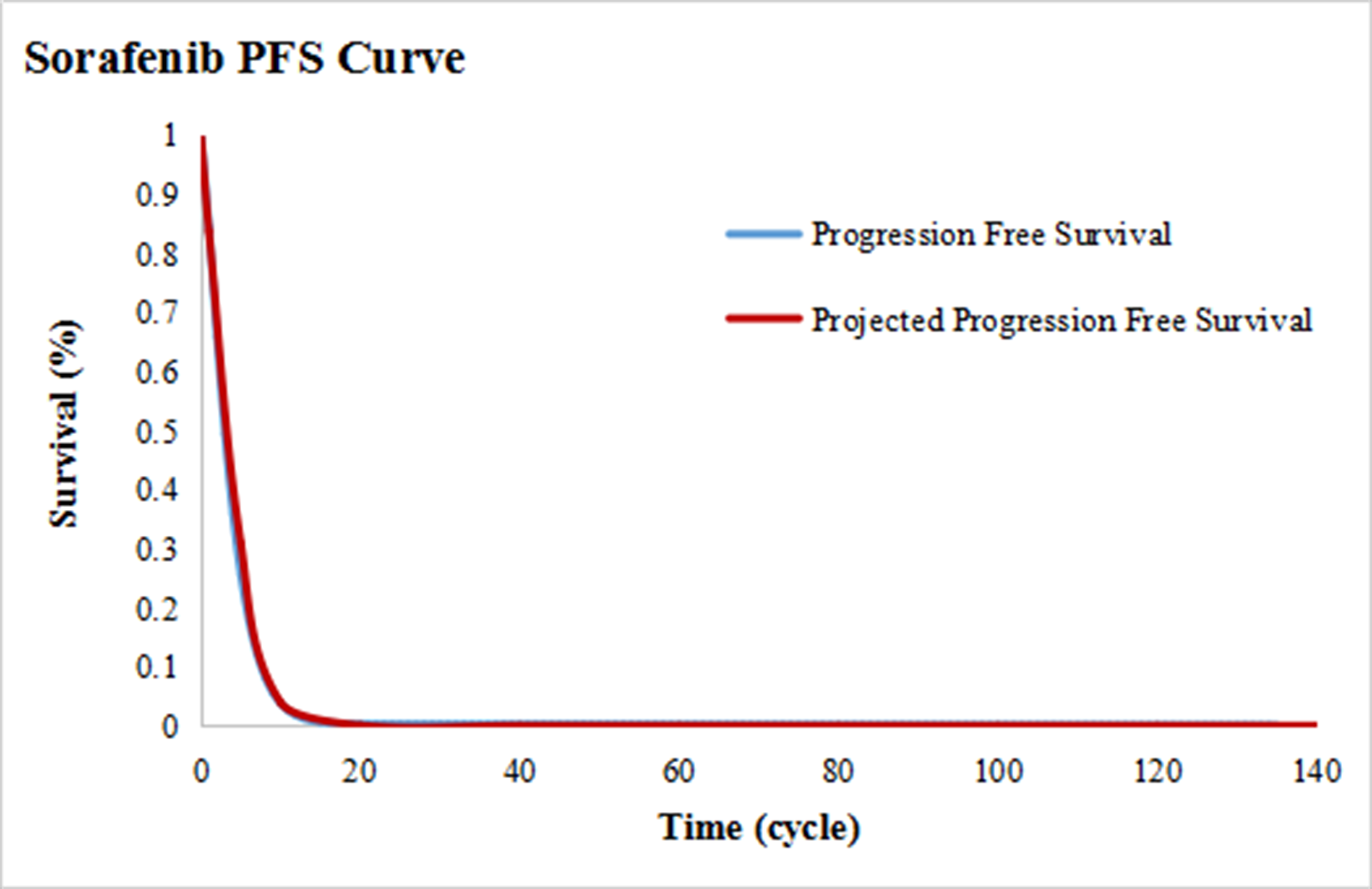

Supplement: Supplementary file 6 [file Image_6.tif]
