# Supplementary material for: Cost-Effectiveness Analysis of Hepatic Arterial Infusion of FOLFOX Combined Sorafenib for Advanced Hepatocellular Carcinoma With Portal Vein Invasion
Source: Front Oncol. 2021 Mar 9;11:562135. doi: 10.3389/fonc.2021.562135 (PMC7985441; doi:10.3389/fonc.2021.562135)
Supplement: Supplementary Table 1 — The cost-effective probability in Monte Carlo 10,000 simulations in different GDP per capita. GDP, Gross Domestic Product. [file Table_1.docx]

Supplemental Table 1: The cost-effective probability in Monte Carlo 10,000 simulations in different GDP per capita.

| **Region** | **WTP**  **(3*GDP per capita, $)** | **Probability of**  **Cost-Effective (%)** |
| --- | --- | --- |
| China | 30492 | 0 |
| Beijing | 72000 | 38.8 |
| Fujian | 47285 | 0.3 |
| Gansu | 14595 | 0 |

GDP: Gross Domestic Product
